# The Relationships Between Suicidal Ideation, Meaning in Life, and Affect: a Network Analysis

**DOI:** 10.1007/s11469-023-01019-9

**Published:** 2023-02-07

**Authors:** Zhihua Guo, Tianqi Yang, Yang He, Wenqing Tian, Chaoxian Wang, Yinling Zhang, Jianjun Liu, Xufeng Liu, Xia Zhu, Shengjun Wu

**Affiliations:** 1 Department of Military Medical Psychology, Air Force Medical University, No. 169 West Changle Road, Xi’an, Shaanxi Province, 710032 China; 2 94995 Troops of People’s Liberation Army, Beijing, China; 3 Department of Nursing, Air Force Medical University, Xi’an, China; 4 Outpatient Department, PLA Air Force 986 Hospital, Xi’an, China

**Keywords:** Suicidal ideation, Meaning in life, Positive affect, Negative affect, Network analysis

## Abstract

Transitioning from holistic analysis to a fine-grained level analysis may provide further understanding of psychopathology. This study aimed to explore dimension-level relationships between suicidal ideation, meaning in life, and affect in a joint framework using network analysis and to identify potential prevention and intervention targets to address suicidal ideation. A total of 852 healthy adults aged 18–35 years completed self-report scales to assess suicidal ideation, meaning in life, and affect. A regularized partial correlation network was then built to examine the links between these dimensions. Expected influence and bridge expected influence values were calculated for each node. The prevalence of suicidal ideation was 4.2%. The search for and presence of meaning in life and positive and negative affect exhibited distinct and complex links to the three dimensions of suicidal ideation (pessimism, sleep, and despair). The important central nodes were search for meaning in life, sleep, despair, and positive affect, while the critical bridge nodes were positive affect, negative affect, and presence of meaning in life. These findings provide further understanding of the specific roles of meaning in life and affect in suicidal ideation. The identified nodes may be promising targets for prevention and intervention for suicidal ideation.

Suicide is an important health problem and a leading cause of death worldwide (Cumming, 2022; Wei et al., 2018). Prior to the COVID-19 pandemic, suicide was the 10th leading cause of death in the USA for all ages, the second leading cause of death for people aged 10–34, and the fourth for ages 34–54 (Hedegaard et al., 2020, 2021). In Europe, central Asia, Australasia, southern Latin America, and high-income Asia Pacific, suicide was also among the top 10 leading causes of death (Naghavi & Global Burden of Disease Self-Harm Collaborators, 2019). A study on suicide rates in China from 1995 to 1999 found that suicide was the fifth major cause of death for all ages and the leading cause of death for young adults aged 15–34 years (Phillips et al., 2002); as of 2010, suicide was the 10th leading cause of death in China (Sun & Zhang, 2015). Due to its tremendous contributions to premature mortality and the associated socioeconomic burden, suicide has attracted increasing concern.

Given its close relationship with suicide, suicidal ideation is of intense interest to suicide researchers. Suicidal ideation, which is defined as thoughts ranging from vague ideas of committing suicide to plans of behaviors intended to end one’s life, is known to be a crucial precursor to suicide (Brown et al., 2000; Crandall et al., 2006; Ramirez Arango et al., 2020; Timpka et al., 2021). Some researchers hold that suicidal ideation is a major phase preceding attempted and completed suicide (Park et al., 2010), and is closely related to subsequent suicide attempts and completed suicide (Coentre & Gois, 2018; Wei et al., 2018). Notably, almost 95% of patients with major depressive disorder (MDD) attempting suicide report suicidal ideation (Sokero et al., 2003). It has also been reported that people who report having had suicidal ideation in the past 12 months are more susceptible to suicide attempts within the following 12 months (Turecki & Brent, 2016). A meta-analysis revealed that higher suicidal ideation is always concomitant with a higher risk for completed suicide in both psychiatric and non-psychiatric populations (Hubers et al., 2018).

Many studies have demonstrated that suicidal ideation is an important predictor and risk factor for completed suicide and increased suicide mortality (Batterham et al., 2013; DeBeer et al., 2016; Hubers et al., 2018; Large et al., 2021; Velupillai et al., 2019). To prevent suicide, substantial efforts should be made to reduce suicidal ideation. To achieve this, studies are needed to investigate the pathological mechanisms underlying the development and maintenance of suicidal ideation, which will result in suicide if not effectively addressed. In other words, it is imperative to examine the variables closely associated with suicidal ideation and the specific relationships between these variables and suicidal ideation in order to effectively manage the development of suicidal ideation, in turn preventing suicide. Among all variables related to suicidal ideation, psychological variables are of particular importance because they have the potential to be altered (Campos et al., 2019; S. T. Liu et al., 2020b).

Meaning in life has been identified as an important protective factor against suicidal ideation; in contrast, loss of meaning in life is a major risk factor for suicidal ideation (Marco et al., 2017; Sun et al., 2021; Whitlock et al., 2013). Meaning in life refers to “the sense made of, and significance felt regarding, the nature of one’s being and existence” and is divided into two dimensions: search for meaning in life and presence of meaning in life (Steger et al., 2006). Increasing research supports the link between meaning in life and suicidal ideation. Individuals with low meaning in life have higher suicidal ideation compared with those with high meaning in life (Harlow et al., 1986; Marco et al., 2017; Steger et al., 2006). Previous studies have shown that meaning in life may act as a buffer against various factors contributing to suicidal ideation, such as bullying victimization, psychological strain, hopelessness, and thwarted belongingness (Beach et al., 2021; Henry et al., 2014; Liu et al., 2020a). Of these two dimensions, presence of meaning in life may be the primary factor for preventing suicide while search for meaning in life may predict or be unrelated to suicidal ideation (Kleiman & Beaver, 2013; Lew et al., 2019). Overall, meaning in life (especially its absence) is tightly associated with suicidal ideation.

Affect, which comprises positive and negative affect (Russell, 2003; Watson et al., 1988), is another psychological variable closely related to suicidal ideation (Cha et al., 2018; Craske et al., 2019; Lucht et al., 2022; Rubio et al., 2020; Yang et al., 2021). Many studies have revealed that negative affect and positive affect, respectively, have significantly positive and negative associations with suicidal ideation (Bennardi et al., 2019; Chabrol et al., 2007; Rubio et al., 2020). A randomized clinical trial found that enhancing positive affect was an effective and practical intervention against suicidal ideation (Craske et al., 2019). Another study reported a bidirectional relationship between suicidal ideation and positive affect (Tian et al., 2017). Thus, positive and negative affect may be relevant indicators for understanding the mechanisms underlying the development of suicidal ideation and potential targets for prevention and intervention. A relationship between meaning in life and positive and negative affect has also been reported in some previous studies. Overall, meaning in life is positively correlated with positive affect but negatively correlated with negative affect (Jin et al., 2016; Liu & Gan, 2010), and positive affect is a strong predictor of meaning in life and may predispose individuals to feel that life is meaningful (King et al., 2006).

As described above, the relationships between suicidal ideation and meaning in life or affect have been thoroughly investigated. In some studies, meaning in life and affect are regarded as two-dimensional variables when examined in relation to suicidal ideation (Bennardi et al., 2019; Kleiman & Beaver, 2013; Lew et al., 2019; Rubio et al., 2020; Yang et al., 2021). These studies commonly investigated the relationships between meaning in life or affect and suicidal ideation with a latent variable approach, using sum scores rather than individual dimension scores to measure suicidal ideation. This practice ignores the heterogeneity of distinct dimensions, obscures the significance of different dimensions, and masks fine-grained relationships between the dimensions of these psychological variables (Fried, 2015; Fried et al., 2016). This practice has hindered understanding the potential pathological pathways between psychopathological variables and identifying targets for more effective intervention (Fried and Nesse, 2015a, b). It has been suggested that the analysis of individual dimensions or symptoms can provide a way forward that could not be discovered by relying solely on aggregate scores (Fried and Nesse, 2015a, b; Liang et al., 2022). Moreover, the dimension-level relationships among meaning in life, affect, and suicidal ideation have never been investigated using a joint framework. This interesting but unexplored question is of significance for further understanding fine-grained links among these variables.

In the present study, we conducted a network analysis of dimension-level meaning in life, affect, and suicidal ideation. Network analysis is an emerging data-driven approach to examine and visualize interactions between symptoms or non-symptoms (Beard et al., 2016; Borsboom, 2017; Borsboom and Cramer, 2013; Guo et al., 2022a). From a network theory perspective, mental disorders emerge from active interactions between symptoms or non-symptoms, rather than just passive reflections of latent variable (Borsboom, 2017; McNally, 2016). According to previous studies (Borsboom, 2017; Epskamp et al., 2018a), the dimensions of psychopathological constructs are represented as nodes, and the interactions between different dimensions are depicted as edges using network analysis. Thus, network analysis is conducive to investigating the specific pathways that link the dimensions of meaning in life or affect with suicidal ideation dimensions. This approach is distinct from the latent variable model and will shed new light on the underlying mechanisms of the formation and maintenance of suicidal ideation. Network analysis also provides centrality indices to determine central nodes that activate all other nodes and exert great influence on the overall network (Borsboom, 2017; Byrne et al., 2021; Guo et al., 2022a), and assesses bridge centrality indices to determine bridge nodes that are critical to maintaining the co-occurrence of variables and transmitting the influence of one variable on another (Guo et al., 2022a, b; Jones et al., 2021; Yuan et al., 2022). To date, the dimension-level network of meaning in life, affect, and suicidal ideation has not been studied.

To fill this research gap, we constructed a network structure of meaning in life, affect, and suicidal ideation and then examined the characteristics of the network. The main aims of this study are threefold: (1) to investigate the connections between meaning in life or affect dimensions and suicidal ideation dimensions; (2) to identify the critical central nodes that influence the entire network; and (3) to identify the critical bridge nodes that facilitate the transmission of positive or negative impact of meaning in life or affect on suicidal ideation. Based on the findings, we attempt to provide theoretical insights into the specific pathological pathways between meaning in life or affect and suicidal ideation, and to provide implications for clinical suicide prevention and intervention.

## Methods

### Study Design and Participants

This study was conducted via an online survey hosted on the Wenjuanxing platform (www.wjx.cn). This study was approved by the Ethics Committee of Xijing Hospital of the Air Force Medical University and performed in accordance with the Declaration of Helsinki.

A total of 900 adults aged 18 years and older were recruited through convenience sampling from May 2022 to June 2022. The inclusion criteria were as follows: (1) healthy adults based on self-report; (2) no self-reported history of neurological or psychiatric illnesses; and (3) able to consent to participate in the study. The exclusion criteria were as follows: (1) incorrect basic information provided in the questionnaire; (2) incomplete questionnaires; and (3) a score ≥ 4 on the concealment dimension of the Self-rating Idea of Suicide Scale. After applying these criteria, the final sample was 852 participants.

### Measures

#### Positive and Negative Affect Scale (PANAS)

The PANAS is a 20-item scale measuring positive and negative affect (Watson et al., 1988). The validated Chinese version of PANAS was used (Huang et al., 2003). Each item is rated on a 5‐point Likert scale from 1 = *very slightly or not at all* to 5 = *extremely*, with higher scores suggesting stronger feelings and emotions. Cronbach’s *α* coefficients of the positive affect and negative affect scales in the present study were 0.93 and 0.92, respectively.

#### Chinese Meaning in Life Questionnaire (C-MLQ)

The C-MLQ is a 10-item scale validated to measure subjective perceptions about life meaning (Steger et al., 2006; Wang & Dai, 2008). There are two dimensions: presence of meaning in life and search for meaning in life. Each dimension is measured by five items rated on a 7-point Likert scale, ranging from 1 = *absolutely untrue* to 5 = *absolutely true*, with higher scores suggesting higher levels of meaning in life. Cronbach’s *α* coefficient of the scale in the present study was 0.85.

#### Self-Rating Idea of Suicide Scale (SIOSS)

The SIOSS is a self-rating scale evaluating suicide ideation (Cheng et al., 2022; Xia et al., 2012). It comprises 26 items divided into 4 dimensions: optimism, sleep, despair, and concealment. Each item is scored as 1 = *yes* or 0 = *no*. Participants with a concealment dimension score ≥ 4 should be excluded. If the total score of despair, optimism, and sleep dimensions is ≥ 12, the participant is considered to have suicidal ideation, with higher scores indicative of stronger suicidal ideation. For convenience of understanding, the optimism dimension is referred to as the pessimism dimension; no other additional alterations were made. Cronbach’s *α* coefficient of the scale in the present study was 0.80.

### Statistical Analysis

We used SPSS22.0 software to calculate the means, standard deviation (SD), and Cronbach’s *α* coefficients. RStudio software (version 4.1.1) was used for network model building and calculation of the expected influence (EI) and bridge expected influence (BEI).

The function *goldbricker* of the R package *networktools* was used to assess potential node redundancy (Jones, 2022); node pairs with strong correlations (*r* ≥ 0.70) and < 20% unique correlations with other nodes are suspected to be redundant (Everaert & Joormann, 2019). The R package *qgraph* was used to build and visualize the network of meaning in life, affect, and suicidal ideation (Epskamp et al., 2012). The network was estimated via the Gaussian graphical model (GGM) (Epskamp et al., 2018b). In network building, the combined use of least absolute shrinkage and selection operator (LASSO) regularization and the extended Bayesian information criterion (EBIC) can attenuate trivial edges to zero, so as to obtain a clear network (Chen & Chen, 2008; Epskamp & Fried, 2018; Foygel & Drton, 2010). We set the EBIC hyperparameter to 0.5 to determine the optimal network model (Epskamp & Fried, 2018; Foygel & Drton, 2010). In the model, nodes represent dimensions and were divided into a meaning in life and affect community and a suicide ideation community; each edge represented the partial correlation between two nodes, with interference from other nodes in the network eliminated by statistical controls (Epskamp & Fried, 2018). Furthermore, we used the R package *bootnet* to test the accuracy of the edge weight and difference in node pairs (Epskamp et al., 2018a). The 95% confidence interval (CI) was computed by non-parametric bootstrapping (1000 bootstrapped samples) to test the accuracy of the edge weight; a relatively narrow CI suggests acceptable accuracy of the edge weight estimation (Mullarkey et al., 2019). We also tested the edge weight difference of node pairs by bootstrapping (*α* = 0.05, 1000 bootstrapped samples).

The R packages *qgraph* and *networktools* were used to calculate the EI and BEI values of each node, respectively (Epskamp et al., 2012; Jones et al., 2021). Node EI is the sum of all edge weights between a given node and other nodes in the network (Robinaugh et al., 2016). Node BEI is the sum of the edge weights between a given node and nodes in another community (Jones et al., 2021). Subsequently, we used the R package *bootnet* to test the difference of node EIs and BEIs and estimate the stability of node EI and BEI (Epskamp et al., 2018a). The difference of node EIs and BEIs was tested by bootstrapping (*α* = 0.05, 1000 bootstrapped samples). The stability of EI and BEI was assessed by case-dropping bootstrapping (1000 bootstrapped samples). We quantified stability using the correlation stability (CS) coefficient, with a value > 0.5 representing ideal stability (Epskamp et al., 2018a).

## Results

### Descriptive Statistics

For the 852 participants, the average age was 23.93 ± 4.17 years (M ± SD, range = 18–35 years) and the majority were male (*n* = 802, 94.1%). Thirty-six (4.2%) participants reached the threshold of having suicide ideation. The means, SDs, EIs, and BEIs of the affect, meaning in life, and suicidal ideation dimensions are shown in Table 1.

**Table 1:** The means, SDs, EIs, and BEIs of each dimension

Variables	M	SD	EI	BEI
Affect				
A1: Positive affect	34.26	7.40	− 0.18	− 0.41
A2: Negative affect	19.45	6.57	0.11	0.28
Meaning in life				
M1: Presence of meaning in life	28.80	5.55	0.06	-0.31
M2: Search for meaning in life	25.20	8.04	0.50	0.07
Suicidal ideation				
S1: Pessimism	0.19	0.58	0.01	-0.28
S2: Sleep	0.72	1.07	0.43	-0.01
S3: Despair	1.47	2.60	0.41	-0.10

*M*, mean; *SD*, standard deviation; *EI*, expected influence; *BEI*, bridge expected influence

### Network Construction

**Fig. 1:**
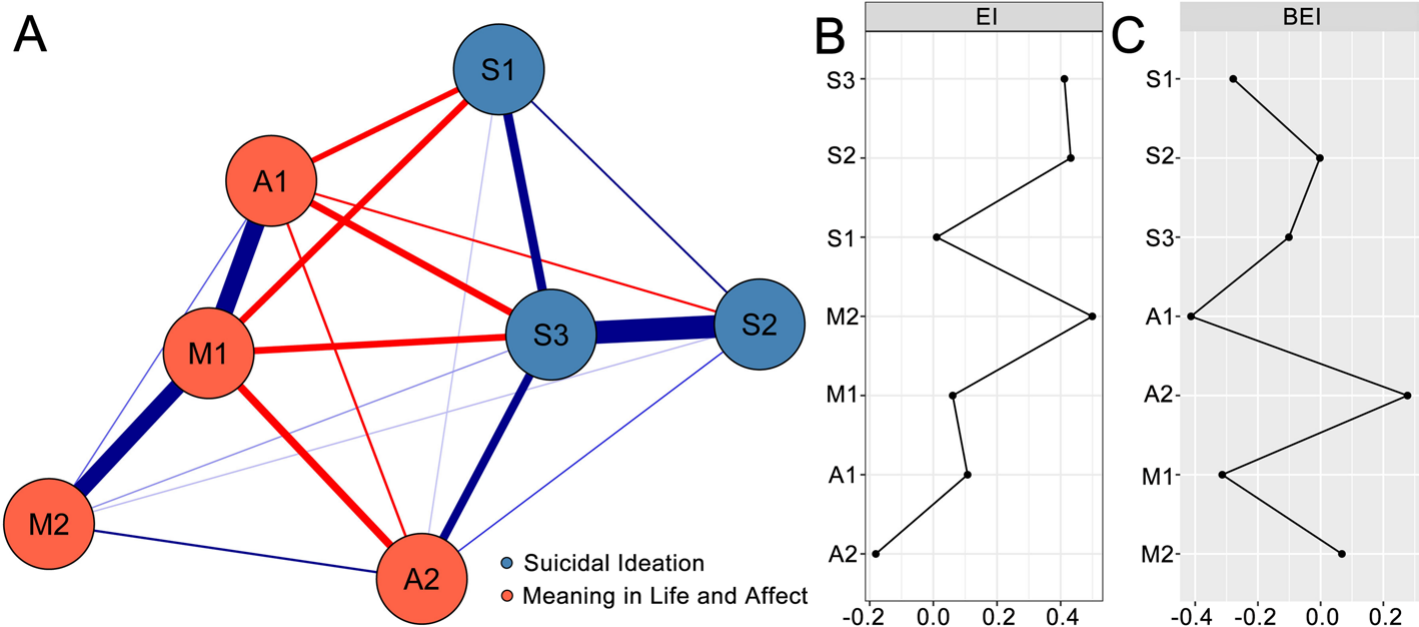
Network model of meaning in life, affect, and suicidal ideation and the EIs and BEIs of the nodes in the network. **A** Network model of meaning in life, affect, and suicidal ideation. The blue line represents a positive partial correlation while the red line represents a negative partial correlation. A thicker line and the more saturated color represents a larger partial correlation coefficient. **B** The EI indices of the nodes in the network (raw values). **C** The BEI indices of the nodes in the network (raw values). A1, positive affect; A2, negative affect; M1, presence of meaning in life; M2, search for meaning in life; S1, pessimism; S2, sleep; S3, despair; EI, expected influence; BEI, bridge expected influence

**Fig. 2:**
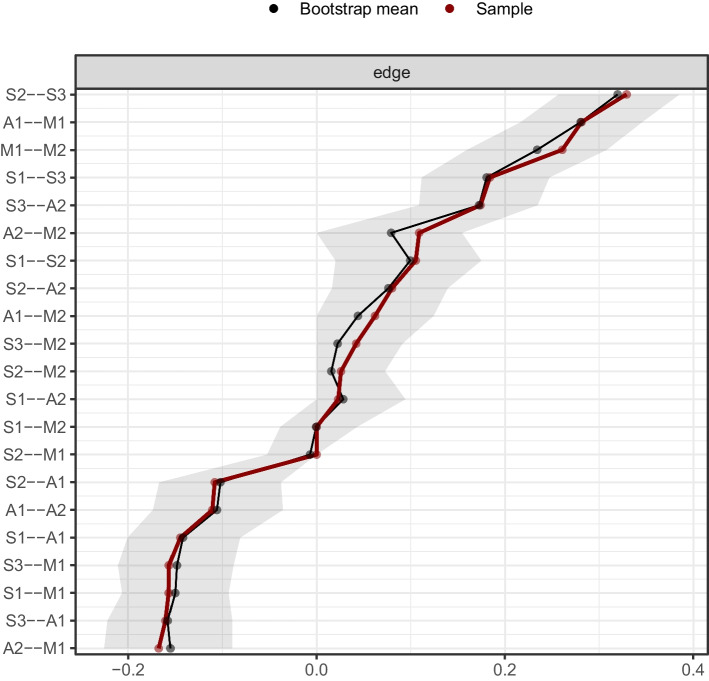
Accuracy of edge weights in the network. The red line depicts the sample edge weights and the gray bar depicts the bootstrapped confidence interval. A1, positive affect; A2, negative affect; M1, presence of meaning in life; M2, search for meaning in life; S1, pessimism; S2, sleep; S3, despair

**Fig. 3:**
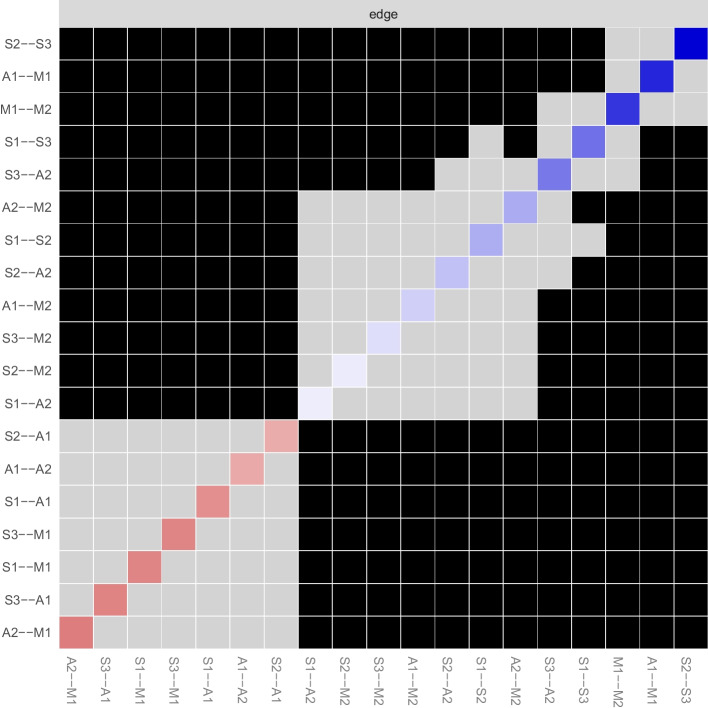
Bootstrapped difference test for edge weights in the network. Gray boxes indicate edge weights that do not differ significantly from one another, while black boxes indicate edge weights that do differ significantly. Blue and red boxes on the diagonal correspond to edge weights with positive and negative correlations, respectively. A1, positive affect; A2, negative affect; M1, presence of meaning in life; M2, search for meaning in life; S1, pessimism; S2, sleep; S3, despair

No potentially redundant nodes were identified. The network model of meaning in life, affect, and suicidal ideation is shown in Fig. 1A. There were 19 edges with non-zero edge weights (ranging from − 0.17 to 0.33) in the network, including nine edges inside the communities and 10 cross-community edges. Of the cross-community edges, there were five positive edges and five negative edges. The positive cross-community edges included A2 “Negative affect”–S3 “Despair” (weight = 0.17) and A2 “Negative affect”–S2 “Sleep” (weight = 0.08), M2 “Search for meaning in life”–S3 “Despair” (weight = 0.04), M2 “Search for meaning in life”-S2 “Sleep” (weight = 0.03), and A2 “Negative affect”-S1 “Pessimism” (weight = 0.02). The negative cross-community edges included A1 “Positive affect”–S3 “Despair” (weight =  − 0.16), M1 “Presence of meaning in life”–S1 “Pessimism” (weight =  − 0.16), M1 “Presence of meaning in life”–S3 “Despair” (weight =  − 0.16), A1 “Positive affect”–S1 “Pessimism” (weight =  − 0.14), and A1 “Positive affect”–S2 “Sleep” (weight =  − 0.11). Some relatively strong edges linked the dimensions of affect and meaning in life, such as M1 “Presence of meaning in life”–A1 “Positive affect” (weight = 0.28), M1 “Presence of meaning in life”–A2 “Negative affect” (weight =  − 0.17), and M2 “Search for meaning in life”–A2 “Negative affect” (weight = 0.11) (see Table 2 for all edge weights of the network model). The 95% CI of edge weights in the network was narrow, indicating that the evaluation of edge weights was accurate (see Fig. 2). The results of the bootstrapped difference test for edge weights are provided in Fig. 3.

**Table 2:** The edge weights in the network model of meaning in life, affect, and suicidal ideation

	S1	S2	S3	A1	A2	M1	M2
S1	0	0.11	0.18	− 0.14	0.02	− 0.16	0.00
S2	0.11	0	0.33	− 0.11	0.08	0.00	0.03
S3	0.18	0.33	0	− 0.16	0.17	− 0.16	0.04
A1	− 0.14	− 0.11	− 0.16	0	− 0.11	0.28	0.06
A2	0.02	0.08	0.17	− 0.11	0	− 0.17	0.11
M1	− 0.16	0	− 0.16	0.28	− 0.17	0	0.26
M2	0	0.03	0.04	0.06	0.11	0.26	0

S1, pessimism; S2, sleep; S3, despair; A1, positive affect; A2, negative affect; M1, presence of meaning in life; M2, search for meaning in life

### Network Centrality

The EI indices of the nodes in the network of meaning in life, affect, and suicidal ideation are shown in Fig. 1B. The results indicate that M2 “Search for meaning in life,” S2 “Sleep,” and S3 “Despair” had the highest positive EI values (EI = 0.50, 0.43, 0.41, respectively). A1 “Positive affect” had the highest negative EI value (EI =  − 0.18). The BEI indices of the nodes are shown in Fig. 1C, which suggest that, in the community of meaning in life and affect, A2 “Negative affect” had the highest positive BEI value (BEI = 0.28). In contrast, A1 “Positive affect” and M1 “Presence of meaning in life” had the highest negative BEI values (BEI =  − 0.41 and − 0.31, respectively).

**Fig. 4:**
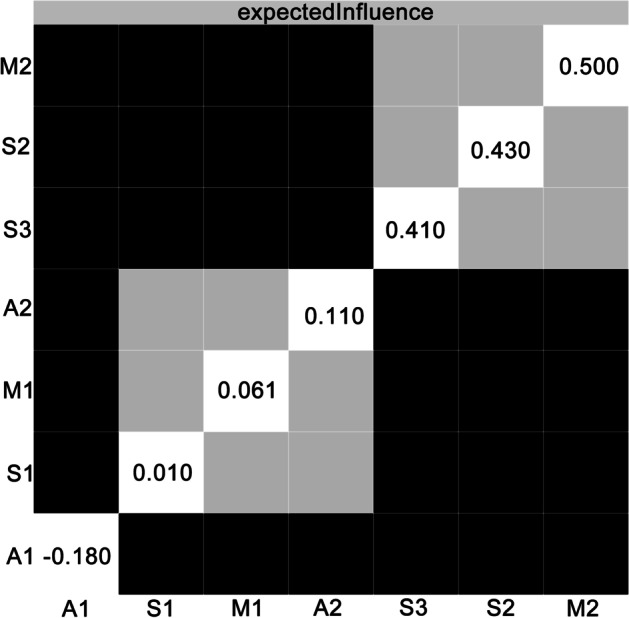
Bootstrapped difference test for node expected influences in the network. Gray boxes indicate node expected influences that do not differ significantly from one another, while black boxes indicate node expected influences that do differ significantly. The numbers in the white boxes (i.e., diagonal line) represent the values of node expected influences. A1, positive affect; A2, negative affect; M1, presence of meaning in life; M2, search for meaning in life; S1, pessimism; S2, sleep; S3, despair

**Fig. 5:**
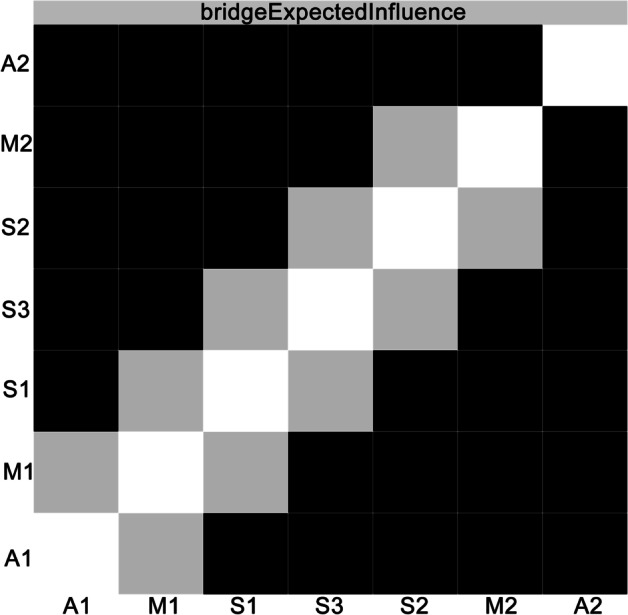
Bootstrapped difference test for node bridge expected influences in the network. Gray boxes indicate node bridge expected influences that do not differ significantly from one another, while black boxes indicate node bridge expected influences that do differ significantly. A1, positive affect; A2, negative affect; M1, presence of meaning in life; M2, search for meaning in life; S1, pessimism; S2, sleep; S3, despair

**Fig. 6:**
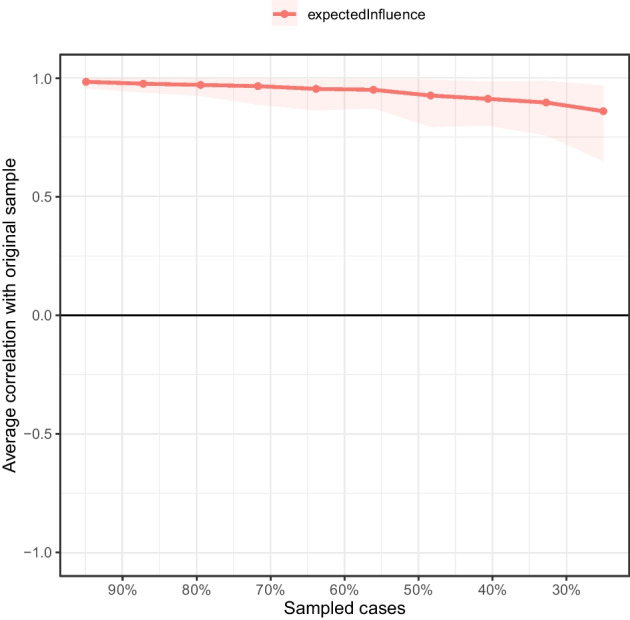
Stability of node expected influences in the network. The red bar represents the average correlation between node expected influences in the full sample and subsample with the red area depicting the 2.5th quantile to the 97.5th quantile

**Fig. 7:**
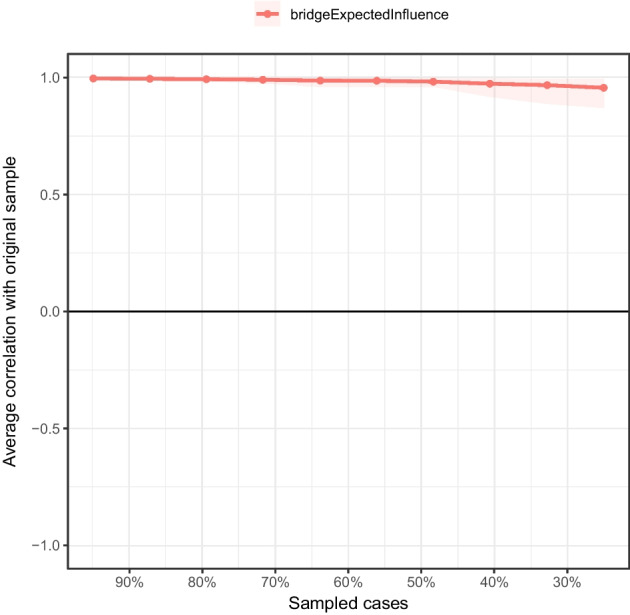
Stability of node bridge expected influences in the network. The red bar represents the average correlation between node bridge expected influences in the full sample and subsample with the red area depicting the 2.5th quantile to the 97.5th quantile

For the current network, the bootstrapped difference test showed that the EI indices of M2 “Search for meaning in life,” S2 “Sleep,” S3 “Despair,” and A1 “Positive affect” were significantly different from those of most other nodes (*P* < 0.05, see Fig. 4). In addition, the BEI indices of A1 “Positive affect,” A2 “Negative affect,” and M1 “Presence of meaning in life” were significantly different from those of most other nodes (*P* < 0.05, see Fig. 5). As suggested by the results of the stability tests of EI and BEI, with a reduction of the sampling proportion, the average correlation with the original sample gently declined (see Figs. 6 and 7). The CS coefficient of EI was 0.67 while that of BEI was 0.75, indicating that the estimations of EI and BEI were both adequately stable.

## Discussion

While numerous studies have investigated the relationships between meaning in life or affect and suicidal ideation (Bennardi et al., 2019; Kleiman & Beaver, 2013), this study is the first to examine dimension-level relationships among them in a joint framework using network analysis. The results revealed that dimensions of meaning in life or affect were connected to dimensions of suicidal ideation, and some of these dimensions were central or bridge nodes that are important for the development and maintenance of suicidal ideation. These findings advance our understanding of the specific pathways underlying the close relations between meaning in life or affect and suicidal ideation, suggesting prevention strategies and interventions against suicidal ideation.

Notably, we found that search for meaning in life had weakly positive associations with the sleep and despair dimensions of suicidal ideation. Another study asserted that search for meaning in life would either predict increased suicidal ideation or be irrelevant to it (Kleiman & Beaver, 2013). It seems reasonable that search for meaning in life is positively related to greater depression symptom severity and lower happiness and life satisfaction (Park, 2010; Wnuk & Charzynska, 2022), and also positively related to negative psychological constructs like rumination and a feeling of a lack of personal growth (Steger et al., 2008). One study found that search for meaning in life is unrelated to suicidal ideation (Lew et al., 2019), while another reported that relationships between search for meaning in life and mental health depended on personal life events (Chen et al., 2021). Hence, relationships between search for meaning in life and suicidal ideation are complex and equivocal, highlighting the need for more studies.

Conversely, the presence of meaning in life was negatively associated with the pessimism and despair dimensions of suicidal ideation. These findings are consistent with previous findings that meaning in life helps prevent suicidal ideation (Kleiman & Beaver, 2013; Lew et al., 2019; Marco et al., 2017). Another study reported that the creation of moments of meaning in life can reduce despair (Attoe & Chimakonam, 2020); individuals who feel confused about meaning in life are predisposed to experience despair and consider suicide (Bowes et al., 2002; Buchanan et al., 1991; Liu et al., 2020b). Moreover, previous studies using multivariate models revealed that meaning in life is significantly positively correlated with optimism, which negatively predicts suicidal ideation (Gravier et al., 2020; O'Keefe & Wingate, 2013). This finding is in line with our result of a negative relationship between presence of meaning in life and pessimism.

We also found that negative affect was positively related to the despair, sleep, and pessimism dimensions of suicidal ideation. This finding is consistent with previous studies concluding that high negative affect is significantly associated with suicidal ideation (Lucht et al., 2022; Rubio et al., 2020; Yang et al., 2021) and the ample evidence of the role of negative affect–related processes increasing the risk of suicidal ideation (Ben-Zeev et al., 2012; Cha et al., 2018). Similarly, a prior network analysis revealed a positive relation between negative affect and suicidal ideation (single-ensemble variable) (Oakey-Frost et al., 2022). In contrast, in the present study, positive affect was negatively associated with all three dimensions of suicidal ideation (pessimism, sleep, and despair). These results are consistent with previous findings that positive affect is a protective factor against suicidal ideation (Brent et al., 2013; Cha et al., 2018; Horwitz et al., 2021; Layron Folgado et al., 2022; Schatten et al., 2021). Furthermore, lower positive affect is prospectively related to suicidal ideation and individuals with suicidal ideation report significant lower positive affect than those without suicidal ideation (Bennardi et al., 2019; Tian et al., 2017).

Additionally, we found connections between the search for and presence of meaning in life and affect, which is consistent with previous studies (Jin et al., 2016; King et al., 2006). Previous studies have reported that search for meaning in life predicts negative affect, while presence of meaning in life is negatively related to negative affect and predicts positive affect (Barnett et al., 2019; Garrosa-Hernández et al., 2013; Liu & Gan, 2010). These close relationships between dimensions of meaning in life and affect highlight their ability to interactively exert indirect influence on suicidal ideation via their respective pathways, indicating they are common factors affecting suicidal ideation and partially explaining why they were intentionally put in one community prior to network analysis.

Based on the EI values, search for meaning in life, sleep, despair, and positive affect was identified as important central nodes, highlighting the impact of these four dimensions in the network. A previous study found that sleep was the central symptom in the suicidal ideation network (Ma et al., 2022), which is partly in line with our findings. However, our findings are inconsistent with those of a study reporting that trait hope and presence of meaning in life were central nodes in the network of suicide cognition, suicidal ideation, and protective factors (Oakey-Frost et al., 2022). This inconsistency likely arises from differences in the scales used and variables included in the network. As our findings are preliminary and largely exploratory, further research is needed to validate them. We also found some bridge nodes; of particular interest are the bridge nodes in the meaning in life and affect community, namely negative affect, positive affect, and presence of meaning in life. Our finding that negative affect was positively linked to all three dimensions of suicidal ideation suggests that negative affect promotes the development of pessimism, sleep, and despair. In contrast, positive affect was negatively linked to these three dimensions and is thus a protective factor. Similarly, presence of meaning in life protects against suicidal ideation.

The findings of this study have important implications both theoretically and clinically. Regarding the theoretical implications, our network analysis examined the interrelationships between suicidal ideation and theoretically derived factors, such as meaning in life and affect. The findings shed light on the detailed pathways connecting search for and presence of meaning in life and positive and negative affect to dimensions of suicidal ideation, thereby providing a fine-grained understanding of the mechanisms underlying these interrelationships. In other words, these findings are of importance to figure out specific role played by different dimensions of meaning in life or affect in the development and maintenance of dimensions of suicidal ideation. Moreover, this study can be considered an important first primary investigation to the identifications of those interconnected variables in a fine-grained level, that may lead to suicidal ideation and then to potential suicide, which may provide ideas and inspiration for similar research in the future. Regarding the clinical implications, central nodes are critical for activating or inhibiting other nodes and contribute greatly to the development and maintenance of the overall network (Borsboom, 2017; Borsboom and Cramer, 2013; Robinaugh et al., 2016), and interventions targeting the central nodes could disrupt the entire network and mitigate the severity of mental problems, facilitating the treatment and prevention (Cai et al., 2022; Guo et al., 2022a; Haws et al., 2022; Liu et al., 2022). In our study, search for meaning in life, sleep, despair, and positive affect were identified as central nodes; therefore, intervention strategies that alleviate search for meaning in life, sleep problems, and despair and strengthen positive affect are potential ways to reduce suicidal ideation. Bridge nodes are critical for understanding mental comorbidities or co-occurring psychopathological constructs. As they transmit the negative or positive impact of one construct to another, they are considered targets for prevention and interventions (Guo et al., 2022b; Huang et al., 2021; Jones et al., 2021; Liang et al., 2022). As negative affect, positive affect, and presence of meaning in life were identified as bridge nodes in our study, they are potential targets for intervention. Preventing the detrimental influence of negative affect and enhancing the protective effect of positive affect and presence of meaning in life could increase the effectiveness of prevention and intervention strategies targeting suicidal ideation. Nowadays, cognitive behavior therapy (CBT) has been an effective treatment that is widely used in the intervention of suicidal ideation (Ecker et al., 2019; Kalmbach et al., 2022; Sinniah et al., 2017; Wu et al., 2022). Our findings indicate that CBT strategies targeting on these identified central and bridge nodes may be of benefit for the prevention and intervention of suicidal ideation. Coincidentally, a recent study has confirmed that CBT for insomnia can prevent and alleviate suicidal ideation for both nonsuicidal and suicidal patients (Kalmbach et al., 2022).

Despite the novel findings of our study, there are several limitations to consider. First, the study used a cross-sectional methodology, which limits the ability to infer causality between dimensions. Future studies are needed to examine causal relationships. Second, the study relied on self-report scales, which may be influenced by recall bias and social approval effects. As a result, we should interpret the findings with caution. Third, although we identified central and bridge nodes that are potential targets for prevention and intervention, longitudinal or experimental research is needed to assess the effectiveness of these clinical strategies. Fourth, the data-driven characteristics of the network approach limit the generalizability of the findings, which mainly focused on healthy male adults. The applicability of our results to other populations requires replication in other samples. Fifth, the network structure constructed in this study reflects between-subject effects at a group level, meaning that it cannot capture idiographic individual-level processes. Finally, the network structure constructed is specific to the scales used and cannot include all dimensions of these constructs. Therefore, the findings provide only preliminary insights. Future studies are encouraged to incorporate other dimensions of the constructs measured using other scales to investigate relations between meaning in life, affect, and suicidal ideation.

## Conclusion

This study is the first to investigate dimension-level interplay between meaning in life, affect, and suicidal ideation in a unified framework using network analysis. Our findings elucidate the specific pathways through which dimensions of meaning in life or affect may interact with dimensions of suicidal ideation. Positive affect and presence of meaning in life are identified as protective factors against suicidal ideation while negative affect and search for meaning in life are as risk factors. The identified central and bridge nodes, which include search for and presence of meaning in life, positive and negative affect, sleep, and despair, suggest prevention and intervention targets against suicidal ideation.

## Data Availability

The raw data supporting the conclusions of this article will be made available by the authors, without undue reservation.
